# Polygenic risk scores for eGFR are associated with age at kidney failure

**DOI:** 10.1007/s40620-025-02207-7

**Published:** 2025-03-03

**Authors:** Kane E. Collins, Edmund Gilbert, Vincent Mauduit, Pukhraj Gaheer, Elhussein A. E. Elhassan, Katherine A. Benson, Shohdan Mohamad Osman, Claire Hill, Amy Jayne McKnight, Alexander Peter Maxwell, Peter J. van der Most, Martin H. de Borst, Weihua Guan, Pamala A. Jacobson, Ajay K. Israni, Brendan J. Keating, Graham M. Lord, Salla Markkinen, Ilkka Helanterä, Kati Hyvärinen, Jukka Partanen, Stephen F. Madden, Joshua Storrar, Smeeta Sinha, Philip A. Kalra, Matthew B. Lanktree, Sophie Limou, Gianpiero L. Cavalleri, Peter J. Conlon

**Affiliations:** 1 School of Pharmacy and Biomolecular Sciences, Royal College of Surgeons in Ireland, Dublin, Ireland; 2 The Science Foundation Ireland FutureNeuro Centre of Excellence, Dublin, Ireland; 3 SFI Centre for Research Training in Genomics Data Science, University of Galway, Galway, Ireland; 4 Nantes University, Ecole Centrale Nantes, INSERM, Center for Research in Transplantation and Translational Immunology, UMR1064, Nantes, France; 5 Division of Nephrology, Departments of Medicine and Health Research Methodology, Evidence and Impact, St. Joseph’s Healthcare Hamilton, McMaster University and Population Health Research Institute, Hamilton, ON Canada; 6 Department of Nephrology and Transplantation, Beaumont Hospital, Dublin, Ireland; 7 Department of Medicine, Royal College of Surgeons in Ireland, Dublin, Ireland; 8 Centre for Public Health, Queen’s University Belfast, Belfast, UK; 9 Department of Epidemiology, University of Groningen, University Medical Center Groningen, Groningen, The Netherlands; 10 Department of Internal Medicine, Division of Nephrology, University of Groningen, University Medical Center Groningen, Groningen, The Netherlands; 11 Division of Biostatistics and Health Data Science, School of Public Health, University of Minnesota, Minneapolis, MN USA; 12 Experimental and Clinical Pharmacology, College of Pharmacy, University of Minnesota, Minneapolis, MN USA; 13 University of Texas Medical Branch, Galveston, TX USA; 14 Department of Surgery, Perelman School of Medicine, University of Pennsylvania, Philadelphia, PA USA; 15 School of Immunology and Microbial Sciences, University College London, London, UK; 16 Finnish Red Cross Blood Service, Research and Development, Biomedicum 1, Helsinki, Finland; 17 Helsinki University Hospital, Transplantation and Liver Surgery, Helsinki, Finland; 18 Data Science Centre, Beaux Lane House, Royal College of Surgeons in Ireland, Dublin, Ireland; 19 Salford Royal Hospital, Northern Care Alliance NHS Foundation Trust, Salford, UK; 20 University of Manchester, Manchester, UK

**Keywords:** Polygenic burden, Age at onset, Kidney failure, Kidney function, Polygenic risk score

## Abstract

**Background:**

The genetic architecture of chronic kidney disease (CKD) is complex, including monogenic and polygenic contributions. CKD progression to kidney failure is influenced by factors including male sex, baseline estimated glomerular filtration rate (eGFR), hypertension, diabetes, proteinuria, and the underlying kidney disease. These traits all have strong genetic components, which can be partially quantified using polygenic risk scores. This paper examines the association between polygenic risk scores for CKD-related traits and age at kidney failure development.

**Methods:**

Genome-wide genotype data from 10,586 patients with kidney failure were compiled from 12 cohorts. Polygenic risk scores for hypertension, albuminuria, rapid decline in eGFR, decreased total kidney volume, and decreased eGFR were calculated using weights from published independent population-scale genome-wide association studies. The association between each polygenic risk score and age at kidney failure was investigated using logistic regression models. The association between polygenic risk score and age at kidney failure was also investigated separately for each primary kidney disease.

**Results:**

Individuals in the highest 10% of polygenic risk score for decreased eGFR developed kidney failure 2 years earlier than those in the bottom 90% (49.9 years and 47.9 years, *P* = 5e-5). A standard deviation increase in decreased eGFR polygenic risk score was associated with increased odds of developing kidney failure before the age of 60 years (Odds ratio (OR) = 1.05; 95% CI 1.01–1.10; *P* = 0.01), as was high decreased eGFR polygenic risk score (OR = 1.26; 95% CI 1.08–1.46; *P* = 0.003).

**Conclusions:**

We conclude that decreased eGFR polygenic risk score explains a portion of the variation in age at development of kidney failure.

**Graphical abstract:**

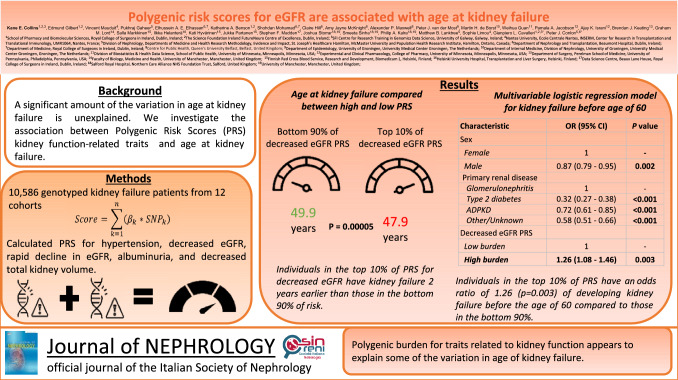

**Supplementary Information:**

The online version contains supplementary material available at 10.1007/s40620-025-02207-7.

## Introduction

Chronic kidney disease (CKD), a condition characterized by reduced estimated glomerular filtration rate (eGFR) and/or proteinuria, is a significant cause of morbidity and mortality on a global scale [1]. The irreversible loss of GFR which CKD causes, irrespective of the primary cause of kidney damage, often leads to kidney failure defined as eGFR below 15 mL/min/1.73 m^2^ or a need for kidney replacement therapy. Kidney failure is a severe, life-limiting condition, where kidney function is critically impaired, and patients require dialysis or transplantation to stay alive. It affects over 800,000 individuals in the United States alone [2], resulting in enormous suffering, premature death, and financial cost. Risk factors, such as male sex, hypertension, diabetes, proteinuria, family history of CKD, lower eGFR, and genetic factors contribute to the progression of CKD [3].

Different causes of CKD can lead to kidney failure at different ages. Factors which have been associated with age at kidney failure include degree of proteinuria, total kidney volume, baseline eGFR and eGFR change [4]. In addition, therapeutic strategies exist to delay the age at which kidney failure develops. These focus on control of hypertension and blood glucose, through diet and lifestyle factors (e.g. exercise), or medications (e.g. ACE and SGLT2 inhibitors, or specific immunosuppressive medications for certain kidney conditions such as glomerulonephritis) [5, 6].

Although inherited monogenic forms of kidney diseases are estimated to account for as much as 70% of the overall prevalence of kidney failure in children and 10–15% in adults [7], the complex polygenic architecture of CKD, which interacts with environmental factors, influences disease progression [8]. Estimates of the heritability of CKD range between 36 and 75% [9].

A recent study with more than one million individuals has enabled the discovery of over 250 loci significantly associated with CKD [10]. Further, well-powered genome-wide association studies (GWAS) have been conducted for diverse traits related to incident CKD and disease progression to kidney failure, including hypertension [11], eGFR [10], albuminuria [12], total kidney volume [13], and rapid decline in eGFR [14]. Summary statistics from genome-wide association studies can be used to calculate polygenic risk scores (PRSs). These scores estimate the cumulative impact of common genetic variations on an individual’s disease status, weighted by the estimated effect size [15]. For instance, individuals in the top 2% of polygenic risk score for eGFR have been reported to be at a threefold greater risk of developing CKD, relative to those in the bottom 98% of polygenic risk score [16]. In a cohort of more than 11,000 participants over the age of 70, it was shown that a standard deviation increase in eGFR polygenic risk score was associated with a decrease of 3.9 mL/min/1.73 m^2^ [17].

Moreover, the polygenic burden for CKD has been reported to alter the penetrance of monogenic kidney disease. Those with autosomal dominant polycystic kidney disease (ADPKD) in the top tertile of polygenic burden for CKD have a significantly higher CKD risk than those in the lowest tertile of polygenic burden [18].

eGFR polygenic risk score has previously been associated with incident CKD stage 3 development [18, 19]. However, testing the association of eGFR polygenic risk score with kidney failure events has been limited to less than a few hundred events [19]. Moreover, the association of polygenic risk score for kidney traits including hypertension, albuminuria, and total kidney volume and age at kidney failure has not been evaluated. Using data from 10,586 patients with kidney failure, we hypothesize that the polygenic burden for CKD progression is associated with age at kidney failure.

## Materials and methods

### Study design and inclusion criteria

To investigate the impact of polygenic burden for traits related to kidney function on age at kidney failure, we assembled a large cohort of patients with kidney failure (*n* = 10,586).

The inclusion criteria were: (1) progression to kidney failure, defined as an eGFR of less than 15 mL/min/1.73 m^2^, starting on chronic dialysis, or having a kidney transplant. For the individuals with kidney failure, age at kidney failure was required, and for those who had a kidney transplant, either age at kidney failure or age at first transplant was required; (2) unrelated to all other participants up to and including the level of 3rd degree (calculated using genotype data, see Supplementary Materials); and (3) availability of single nucleotide polymorphism (SNP) array genotyping data.

### Patient cohort descriptions

We collected 10,586 individuals from 12 cohorts from the following countries: Canada—Canadian study of prediction of death, dialysis and interim cardiovascular events (CanPREDDICT), Genetic Epidemiology of CKD Outcomes (GECKO), GECKO—Transplant (GEKCO-T); USA—Deterioration of Kidney Allograft Function (DeKAF), Genomics of kidney transplantation (GEN03); Finland—Finnish Red Cross Blood Service (FRCBS); Netherlands—TransplantLines (TL); France—Kidney Transplantation—Genomic Investigation of Essential Clinical concerns (KiT-GENIE); Ireland—Irish kidney gene project (IKGP), United Kingdom and Ireland Renal Transplant consortium (UKIRTC); UK—Salford Kidney Study (SKS), Queen’s University Belfast (QUB). See Supplementary Materials for more detailed information on the recruitment and characteristics of each of these cohorts involved.

Patients were classified into primary kidney disease groups of glomerulonephritis (including IgA nephropathy), ADPKD, type 2 diabetes, and other/unknown. These groups were chosen as they represented the largest groups of patients, and other groups had too few patients (< 10% of cohort). Other (39%) and unknown (11%) forms of primary kidney disease were grouped together due to the heterogeneous nature of the other forms of primary kidney disease. This group included: hereditary nephropathy, Alport syndrome, congenital renal hypoplasia, interstitial nephritis, congenital renal dysplasia, renal vascular disease, nephrocalcinosis, and kidney tumor. If a patient was a kidney transplant recipient, and only had information on age at kidney transplant (not age at kidney failure directly), then age at transplant was used to calculate estimated age at onset of kidney failure. For such individuals, we used age at transplant minus four years for the North American cohorts and age at transplant minus 2.5 years for the European cohorts as a proxy for age at onset of kidney failure. This was based on the average wait time in the US being 3–5 years [20] and the average time in Europe being 2–3 years [21, 22].

### Polygenic risk score calculation

Genotype data were subject to quality control for minor allele frequency, missingness per marker, and missingness per individual (see Supplementary Materials). We calculated polygenic risk score in each individual for hypertension [11], eGFR [23], rapid decline in eGFR (defined by > 5 ml/min/1.73 m^2^/year) [14], albuminuria [12], and total kidney volume [13] using published data from genome-wide association studies for each trait. Further details of these genome-wide association studies can be found in Supplementary Table S2. Polygenic risk scores were calculated using PRSice2 [24], selecting alleles with a p-value threshold greater than 0.5 (see Supplementary Materials for further details). All analyses were conducted in R, using version 4.2.1 (2022-06-23) [25].

For two of these polygenic risk scores (eGFR and total kidney volume), we hypothesized that higher values would be associated with better kidney function [26], while for the others (hypertension, albuminuria, stroke, intracranial aneurysm, and rapid kidney function decline), one might expect that higher values would be associated with worse kidney function. To simplify interpretation, we standardized the directionality of all polygenic risk scores, such that one might expect higher scores to be associated with negative outcomes. We did this by inverting the sign of the eGFR and total kidney volume polygenic risk scores to create “new” polygenic risk scores, which we will refer to as “decreased eGFR”, and “decreased total kidney volume”.

### Statistical analysis

Patients were split into two groups, “high” polygenic risk score, and “non-high” polygenic risk score, for each of the five polygenic traits for the following thresholds: high polygenic risk score—top 2%, non-high polygenic risk score—bottom 98%, high polygenic risk score—top 5%, non-high polygenic risk score—bottom 95%, high polygenic risk score—top 10%, non-high polygenic risk score—bottom 90%, high polygenic risk score—top 20%, non-high polygenic risk score—bottom 80%. Mean age at kidney failure was compared between the “high” polygenic risk score and the “non-high” polygenic risk score groups for each of these thresholds using the function *t* test.

Data were split into four subgroups for each primary kidney disease (glomerulonephritis, ADPKD, type 2 diabetes and other/unknown). Mean age at kidney failure for each primary kidney disease subgroup was compared between high and non-high polygenic risk score burden (defined as top 10% and bottom 90%) for each trait using a series of *t* tests in R.

Univariable logistic regression models were created using the R function *glm* to test the association between sex, primary kidney disease, and each polygenic risk score trait and the odds of developing kidney failure before the age of 60. The effect of each polygenic risk score was investigated as a continuous variable, and as a discrete variable (discretized into non-high and high burden in the above manner).

Multivariable logistic regression models were then created using the R function *glm* to test the association between the statistically significant factors from the univariate analysis and the odds of developing kidney failure before the age of 60. Separate models were constructed for each continuous and discrete polygenic risk score.

We also conducted power calculations to determine the smallest effect size that could be reliably detected (see Supplementary Materials).

## Results

Table 1 shows the characteristics of the 10,586 patients with kidney failure that passed genotyping Quality Control. The median age at kidney failure was 50 years and there were more males (6676, 63%) than females. One thousand six hundred and ten individuals (15%) had ADPKD, 1756 (17%) had glomerulonephritis, 1329 (13%) had type 2 diabetes, 5891 (56%) had some other or unknown form of kidney disease. Supplementary Table S1 contains patient characteristics split by primary kidney disease. Power calculations (for 95% power and 0.05 significance) showed that the smallest effect size that could be reliably detected is 8.5 months.

**Table 1:** Demographic characteristics of study participants

Cohort	Number of patients	Age at kidney failure (years), median (range)	Female, *n* (%)	Primary renal disease			
				Glomerulonephritis, *n* (%)	Other/unknown, *n* (%)	Autosomal dominant polycystic kidney disease, *n* (%)	Type 2 diabetes, *n* (%)
CanPREDDICT	614	70 (23–94)	217 (35)	83 (14)	247 (40)	47 (8)	237 (39)
DeKAF	1676	48 (0–79)	620 (37)	390 (23)	532 (32)	263 (16)	491 (29)
FRCBS	954	56 (18–77)	297 (31)	259 (27)	445 (47)	170 (18)	80 (8)
GECKO	84	68 (20–93)	39 (46)	7 (8)	45 (54)	6 (17)	26 (31)
GECKO-T	276	50 (1–79)	111 (10)	49 (18)	122 (44)	41 (15)	64 (23)
GEN03	998	47 (1–77)	397 (40)	293 (29)	347 (35)	139 (14)	219 (22)
KiT-GENIE	1831	52 (16–85)	644 (35)	0 (0)	1551 (85)	280 (15)	0 (0)
IKGP	194	50 (25–84)	91 (47)	0 (0)	0 (0)	194 (100)	0 (0)
QUB	150	41 (2–70)	62 (41)	32 (21)	96 (64)	18 (12)	4 (3)
SKS	732	68 (24–95)	246 (34)	61 (8)	415 (57)	89 (12)	167 (23)
TL	1056	48 (16–72)	448 (42)	264 (25)	642 (61)	129 (12)	21 (2)
UKIRTC	2021	44 (16–77)	738 (37)	318 (16)	1449 (72)	234 (12)	20 (1)
Total	10,586	50 (0–95)	3910 (37)	1756 (17)	5891 (56)	1610 (15)	1329 (13)

For further details regarding the recruitment and characteristics of each cohort, see the Supplementary Materials

*CanPREDDICT* Canadian study of prediction of death, dialysis and interim cardiovascular events, *DeKAF* deterioration of kidney allograft function, *FRCBS* finnish red cross blood service, *GECKO* genetic epidemiology of CKD outcomes, *GEKCO-T* GECKO-transplant, *GEN*03 genomics of kidney transplantation, *KiT-GENIE* kidney transplantation-genomic investigation of essential clinical concerns, *IKGP* Irish kidney gene project, *QUB* Queen’s University Belfast, *SKS* Salford kidney study, *TL* transplantlines, *UKIRTC* United Kingdom and Ireland renal transplant consortium

### Influence of polygenic burden on age at kidney failure

To investigate whether polygenic burden influences age at kidney failure, we compared age at kidney failure between those with high polygenic risk score burden for each trait for five thresholds (top 2% vs rest, top 5% vs rest, top 10% vs rest, top 20% vs rest). Out of the five studied polygenic risk score traits, only the decreased eGFR polygenic risk score showed a significant difference between high and non-high polygenic burden (Table 2). Patients in the top 5% and top 20% of decreased eGFR polygenic risk score burden developed kidney failure at a significantly younger age compared to those in the bottom 95% and 80% of burden, respectively (47.9 years versus 49.8 years; *P* = 0.004, 48.8 years vs 50.0 years; *P* = 0.002, respectively, Table 2). Similarly, patients within the top 10% of decreased eGFR polygenic risk score burden developed kidney failure at a significantly younger age compared to the bottom 90% of burden (47.9 years versus 49.9 years; *P* < 0.0005, Table 2, Supplementary figure S1).

**Table 2:** Comparison of age at kidney failure using different definitions of high and non-high PRS burden for each trait

PRS	Threshold	Age at kidney failure (years)		
		High burden	Non-high burden	*P* value
Albuminuria	Top 2% vs bottom 98%	49.5	49.7	0.78
	Top 5% vs bottom 95%	50.1	49.7	0.59
	Top 10% vs bottom 90%	49.5	49.8	0.66
	Top 20% vs bottom 80%	49.3	49.8	0.18
Hypertension	Top 2% vs bottom 98%	48.8	49.8	0.39
	Top 5% vs bottom 95%	48.8	49.8	0.15
	Top 10% vs bottom 90%	49	49.8	0.14
	Top 20% vs bottom 80%	49.5	49.8	0.46
Decreased eGFR	Top 2% vs bottom 98%	49	49.7	0.48
	**Top 5% vs bottom 95%**	**47.9**	**49.8**	**0.004**
	**Top 10% vs bottom 90%**	**47.9**	**49.9**	**0.00005**
	**Top 20% vs bottom 80%**	**48.8**	**50**	**0.002**
Decreased total kidney volume	Top 2% vs bottom 98%	50.3	49.7	0.60
	Top 5% vs bottom 95%	50.3	49.7	0.38
	Top 10% vs bottom 90%	50.1	49.7	0.49
	Top 20% vs bottom 80%	50.2	49.6	0.16
Rapid eGFR decline	Top 2% vs bottom 98%	50.4	50.6	0.88
	Top 5% vs bottom 95%	49	50.7	0.04
	Top 10% vs bottom 90%	49.6	50.8	0.07
	Top 20% vs bottom 80%	50.2	50.7	0.28

*P* values < 0.05 are bolded

There is a difference of between 1.3 and 2 years in age at kidney failure between patients with high and non-high burden for decreased eGFR. Given that we tested 5 traits, a Bonferroni-adjusted significance threshold would be *P* < 0.01

*PRS* polygenic risk score, *eGFR* estimated glomerular filtration rate

We then examined the impact of the high and non-high polygenic burden of the five polygenic risk score traits on the patient’s primary kidney disease. Statistically significant differences were observed in the age at kidney failure and decreased eGFR polygenic risk score burden between glomerulonephritis and type 2 diabetes (Supplementary table S3). Among patients with glomerulonephritis, those in the top 10% of decreased eGFR polygenic risk score developed kidney failure at the age of 41.6 years compared to 45.8 years for those in the bottom 90% of polygenic risk score (*P* = 0.0007). Similarly, for patients with type 2 diabetes as their primary kidney disease, those in the top 10% of decreased eGFR polygenic risk score developed kidney failure at 52.2 years compared to 56.4 years for those in the bottom 90% of polygenic risk score (*P* = 0.002).

To investigate which factors impact age at kidney failure, we created univariable logistic regression models for kidney failure before the age of 60 years. Male sex (Odds ratio (OR) = 0.87; 95% Confidence Interval (CI) 0.79–0.95; *P* = 0.001, Table 3) and primary kidney disease type were both significantly associated with kidney failure development. Decreased eGFR polygenic risk score as a continuous variable was associated with kidney failure before the age of 60 years (OR = 1.05; 95% CI 1.004–1.09; *P* = 0.03, Table 3), but did not withstand a multiple testing correction. The discretized eGFR polygenic risk score has a stronger and more significant association with kidney failure before the age of 60 years (OR = 1.23; 95% CI 1.06–1.43; *P* = 0.006, Table 3). Rapid eGFR decline polygenic risk score did not pass the multiple correction threshold of 0.01 (OR = 1.22; 95% CI 1.03–1.47; *P* = 0.03, Table 3). There was no significant effect of polygenic risk score for hypertension, albuminuria, or decreased total kidney volume burden.

**Table 3:** Univariable logistic regression models investigating the association between kidney failure development before the age of 60 years and sex, primary renal disease, continuous PRS, and discretized PRS

	OR (95% CI)	*P* value
Sex		
Female	1	–
Male	**0.87 (0.79, 0.95)**	**0.001**
Primary renal disease		
Glomerulonephritis	1	–
Other/Unknown	0.59 (0.51, 0.67)	**2e-15**
ADPKD	0.74 (0.62, 0.87)	**2.9e-4**
Type 2 Diabetes	0.32 (0.27, 0.38)	** < 2e-16**
**Continuous PRS**		
Hypertension PRS	0.99 (0.95, 1.03)	0.75
Decreased eGFR PRS	1.05 (1.004, 1.09)	**0.03**
Albuminuria PRS	1.01 (0.97, 1.06)	0.6
Rapid eGFR decline PRS	1.02 (0.97, 1.07)	0.51
Decreased total kidney volume PRS	0.97 (0.93, 1.02)	0.22
**Discrete PRS**		
Hypertension		
Non-high (bottom 90%)	1	–
High (top 10%)	1.03 (0.89, 1.19)	0.71
Decreased eGFR		
Non-high (bottom 90%)	1	–
High (top 10%)	1.23 (1.06, 1.43)	**0.006**
Albuminuria		
Non-high (bottom 90%)	1	–
High (top 10%)	1.15 (0.97, 1.33)	0.06
Rapid eGFR decline		
Non-high (bottom 90%)	1	–
High (top 10%)	1.22 (1.03, 1.47)	0.03
Decreased total kidney volume		
Non-high (bottom 90%)	1	–
High (top 10%)	1.01 (0.88, 1.17)	0.88

*P* values < 0.05 are bolded

Given that we tested five traits, a Bonferroni-adjusted significance threshold would be *P* < 0.01. The odds ratio is the odds of developing kidney failure before the age of 60 years for each factor

*OR* odds ratio, *CI* confidence interval, *PRS* polygenic risk score, *eGFR* estimated glomerular filtration rate

Two separate multivariable logistic regression models were then created to predict kidney failure development before the age of 60 years, both controlling for sex and primary kidney disease, one with the continuous decreased eGFR polygenic risk score, and the other with the discrete one. Male sex and primary kidney disease were both significant in these models, as was the continuous decreased eGFR polygenic risk score (OR = 1.05; 95% CI 1.01–1.10; *P* = 0.01, Table 4), and the discrete decreased eGFR polygenic risk score (OR = 1.26; 95% CI 1.08–1.46; *P* = 0.003, Table 4).

**Table 4:** Multivariable logistic regression models investigating the association between kidney failure development before the age of 60 years and sex, primary renal disease, continuous decreased eGFR PRS, and discretized decreased eGFR PRS

Characteristic	OR (95% CI)	*P* value
**Model 1 (Continuous PRS)**		
Sex		
Female	1	–
Male	0.87 (0.79–0.95)	**0.002**
Primary renal disease		
Glomerulonephritis	1	–
Type 2 diabetes	0.32 (0.27–0.38)	** < 2e-16**
ADPKD	0.72 (0.61–0.85)	**1.3e-4**
Other/Unknown	0.58 (0.50–0.66)	**4e-16**
Decreased eGFR PRS	1.05 (1.01–1.10)	**0.01**
**Model 2 (Discrete PRS)**		
Sex		
Female	1	–
Male	0.87 (0.79–0.95)	**0.002**
Primary renal disease		
Glomerulonephritis	1	–
Type 2 diabetes	0.32 (0.27–0.38)	** < 2e-16**
ADPKD	0.72 (0.61–0.85)	**1.4e-4**
Other/Unknown	0.58 (0.51–0.66)	**5e-16**
Decreased eGFR PRS		
Non-high (bottom 90%)	1	–
High (top 10%)	1.26 (1.08–1.46)	**0.003**

*P* values < 0.05 are bolded

Given that we tested 5 traits, a Bonferroni-adjusted significance threshold would be *P* < 0.01

The odds ratio is the odds of developing kidney failure before the age of 60 years for each factor

*OR* odds ratio, *CI* confidence interval, *PRS* polygenic risk score, *eGFR* estimated glomerular filtration rate

## Discussion

We explored the impact of polygenic burden for kidney traits on age at kidney failure using 12 cohorts comprising 10,586 individuals with kidney failure.

A top 10th percentile polygenic risk score for decreased eGFR was associated with two years earlier age at kidney failure, and increased likelihood of developing kidney failure before the age of 60 years. This effect is seen particularly strongly in those with primary kidney diseases of glomerulonephritis and type 2 diabetes. Importantly, although hypertension and proteinuria are known to be significant risk factors for progression of CKD, we were unable to demonstrate an effect of polygenic risk score for these variables in this large cohort of CKD patients.

A previous study, involving nearly 9000 individuals, assessed the impact of polygenic risk score for eGFR on the risk of developing both CKD and kidney failure and found hazard ratios of 1.30 and 1.20, respectively associated with standard deviation increases in polygenic risk scores [19]. However, only 470 individuals (5%) had kidney failure, significantly less than our over 10,000 individuals with kidney failure. While the impact of polygenic risk score on age at kidney failure in our study is relatively modest (2 years between high and non-high polygenic burden groups), these results align with this previous study. In a separate, cross ancestry study, those in the top 2% of a CKD polygenic risk score were shown to have a nearly threefold increased risk of CKD [16]. This study utilized a different polygenic risk score which was a derivative of the eGFR polygenic risk score but was optimized for studying all global ancestry populations, not just those of European ancestry.

Furthermore, polygenic risk scores provide value in interpreting family history data [27]. Polygenic risk scores have been developed for different kidney disease types and, in particular, they are most advanced for IgA nephropathy where a recent genome-wide association study of more than 10,000 individuals from 17 international cohorts identified 30 loci associated with IgA nephropathy development, and these loci explained 11% of disease risk [28]. In contrast to monogenic disease caused by rare pathogenic variants, where genetic and allelic heterogeneity is the expectation, polygenic scores seek to identify the cumulative impact of common variants of small individual effects which would be expected to contribute to disease progression regardless of the primary etiology. These variants may work through a multitude of pathophysiological processes including, but not limited to, fibrosis, inflammation, metabolic disease, hypertension, and more. Whilst polygenic risk scores typically explain only a fraction of the heritability of a trait, this is likely to improve as genome-wide association study sample sizes increase and methods are developed to integrate rare variants into polygenic risk scores [29]. It is also important to note that the polygenic risk score for rapid eGFR decline was calculated using genome-wide association studies involving 19,000 cases and 175,000 controls, that only found 7 genome-wide significant loci (variance explained not reported), compared to over 260 loci for the eGFR genome-wide association studies, involving over 560,000 individuals and explaining 7.1% of trait variation (Supplementary Table S2). This relative lack of power of the underlying genome-wide association studies may partially explain why we did not find significant associations of age at kidney failure with this rapid eGFR decline polygenic risk score.

A recent study with more than 12,000 multiple sclerosis patients found that the variants associated with disease severity are different from those associated with disease susceptibility [30]. Such a study highlights the need for specific, well-powered genome-wide association studies for kidney failure, thus enabling more powerful kidney failure polygenic risk scores. The genome-wide association study for eGFR, upon which this study is based, was conducted in largely healthy individuals. It is possible that significantly different variants may play a role in a genome-wide association study of kidney failure than a genome-wide association study for eGFR in largely healthy individuals.

Although the effect sizes observed in our study may be considered modest, they are statistically significant and can explain two years difference in age at kidney failure. Furthermore, there was a 26% greater risk of kidney failure before the age of 60 years in the high polygenic risk score group (top 10%) compared to the bottom 90% polygenic risk score group. As genome-wide association studies continue to grow in size and predictive power, polygenic risk score could potentially explain a more substantial proportion of the age at kidney failure, thereby becoming increasingly valuable in clinical decision-making. In such a case, polygenic risk score for decreased eGFR could be one of a number of factors used to aid in risk stratification and personalized treatment decisions (including proactive disease management) for individuals at risk of kidney failure.

Our study has limitations. Age at kidney failure was not known in all individuals and we used proxy ages at kidney failure in some of the cohorts with transplant recipients. Broad categorization of primary kidney diseases also introduced noise and reduced statistical power (the smallest effect size that this study is powered to detect is approximately 8.5 months). Future studies should strive for more precise phenotyping. It is notable that in this cohort, male sex results in a 13% lower risk of developing kidney failure before the age of 60 compared to females. This result is not actually necessarily contradictory to the fact that men are at higher risk of kidney failure than women. We found this in our cohort, in that 65% of the kidney failure cohort is male. Additionally, while our dataset comprises a diverse range of kidney diseases, it is possible that polygenic risk score performance may vary across different kidney disease types. While our cohort provides some evidence of this possibility, more research is required. Other clinical factors that may modify the effect, such as hypertension and diabetes status, were not available and may have improved prediction.

It is apparent from our study that the utility of polygenic risk score in defining renal phenotype appears to be primarily at the extremes of polygenic risk score, i.e., when comparing patients with high polygenic risk score for each variable compared to those with non-high polygenic risk score. Similar effects have also been found by others including one study which reported individuals in the top 2% of CKD polygenic risk score as having a nearly threefold increased risk of CKD compared to those in the bottom 98% [16]. Another large study reported that polygenic risk alters the penetrance of monogenic kidney disease [18]. It is notable that, while our results suggest those in the top 2% have an earlier age at kidney failure than those in the bottom 98% (49 years vs 49.7 years), this is not statistically significant (*P* = 0.48), likely due to a lack of power involved by comparing a group with relatively few individuals.

Given that our dataset exclusively consisted of individuals of European ancestry, caution should be exercised when applying our findings to other populations. Studies like those that create a trans-ancestry polygenic risk score [16] may offer valuable insights into diverse patient groups.

In summary, our meta-analysis unveils the impact of inherited genetic factors associated with kidney function on age at kidney failure. The incorporation of the kidney function-related polygenic risk score into clinical practice may hold promise for risk assessment and treatment strategies. While challenges remain, the growing power of gene wide association studies and polygenic risk score warrants further investigation into their potential utility in improving patient outcomes and informing transplantation decisions. This is made increasingly feasible by the ever-decreasing cost of single nucleotide polymorphism-array genotyping, which now costs in the region of $50 per sample [31]. These results suggest that genetic factors related to kidney function play a role in determining the timing of kidney failure. Our findings provide novel insights into the genetic determinants of kidney failure onset and have potential implications for clinical practice and future research.

## Supplementary Information

Below is the link to the electronic supplementary material.

Supplementary file1 (DOCX 31 KB)

## Data Availability

The data used in this study are not publicly available due to concerns regarding patient confidentiality and privacy. Access to the data can be requested from each participating center individually, subject to their respective data sharing policies and ethical considerations.
