# Supplementary material for: Polygenic risk scores for eGFR are associated with age at kidney failure
Source: J Nephrol. 2025 Mar 3;38(3):969–78. doi: 10.1007/s40620-025-02207-7 (PMC12165877; doi:10.1007/s40620-025-02207-7)
Supplement: Supplementary file 1 — Supplementary file1 (DOCX 31 KB) [file 40620_2025_2207_MOESM1_ESM.docx]

# Contents

[**Contents 1**](#_gjdgxs)

[Data characteristics 2](#_30j0zll)

[Description of cohorts 2](#_1fob9te)

[Summary statistics by primary renal disease 5](#_3znysh7)

[Power analysis 5](#_2et92p0)

[**Data QC and processing 7**](#_tyjcwt)

[**Details of GWAS used to generate PRS 8**](#_1t3h5sf)

[Validation of PRS methods 9](#_4d34og8)

[PRS as a modifier of PKD primary mutation 10](#_2s8eyo1)

[Bibliography 12](#_17dp8vu)

# Data characteristics

## Description of cohorts

We assembled 13 different cohorts for this study, all of European ancestry:

**Canadian study of prediction of death, dialysis and interim cardiovascular events (CanPREDDICT)** is a prospective Canadian study that investigated renal disease progression in individuals with a baseline eGFR between 15 - 45[^1^](https://www.zotero.org/google-docs/?utBHL6). It contains 641 genotyped individuals of European ancestry.

**Genetic Epidemiology of CKD Outcomes (GECKO)** is a study that recruited patients approaching or diagnosed with ESRD. 86 Canadian individuals[^2^](https://www.zotero.org/google-docs/?MNK5dX).

**GECKO – Transplant (GEKCO-T)** is a study that recruited kidney transplant recipients. 276 Canadian individuals[^2^](https://www.zotero.org/google-docs/?rY5pDg).

**Deterioration of Kidney Allograft Function (DeKAF) and GEN03:** The Deterioration of Kidney Allograft Function (DeKAF) and GEN03 studies are cohorts of transplant recipients from 7 transplant centres in the US and Canada[^3^](https://www.zotero.org/google-docs/?jBYd21)^,^[^4^](https://www.zotero.org/google-docs/?9gVCbP). They contributed 1,676 and 998 transplant recipients who all had kidney failure, respectively, to this study.

**Finnish Red Cross Blood Service (FRCBS):** 929 genotyped transplant recipients transplanted in a single centre in Helsinki between 2007 and 2017[^5^](https://www.zotero.org/google-docs/?lCGgFQ).

**Kidney Transplantation - Genomic Investigation of Essential Clinical concerns (KiT-GENIE):** 1,831 transplant recipients were collected from the French monocentric KiT-GENIE cohort[^6^](https://www.zotero.org/google-docs/?Dh0NhR).

**Mayo clinic PKD cohort (Mayo)**: 3489 genotyped patients with PKD from the Mayo clinic[^7^](https://www.zotero.org/google-docs/?Hvt5t2).

**Queen’s University Belfast (QUB):** 150 genotyped transplant recipients from across Northern Ireland. These samples were distinct from the samples included in the UKIRTC[^8^](https://www.zotero.org/google-docs/?LfswtX).

**Irish kidney gene project (IKGP):** 356 genotyped patients with PKD from across Ireland[^9^](https://www.zotero.org/google-docs/?GuqOkl).

**Salford Kidney Study (SKS):** 771 genotyped British patients with kidney failure from a prospective, longitudinal, study of more than 3,000 patients with CKD[^10^](https://www.zotero.org/google-docs/?u6b9AN).

**Transplant Lines (TL):** 1,056 genotyped transplant recipients from the Netherlands. This cohort is a single-centre biobank from the University Medical Centre Groningen including all different types of solid organ transplant recipients and living organ donors[^11^](https://www.zotero.org/google-docs/?ckm0iW). These individuals were imputed using the 1000 Genomes imputation panel.

**United Kingdom and Ireland Renal Transplant consortium (UKIRTC):** 2,032 genotyped transplant recipients from all kidney transplant centres in the UK and Ireland that took place between 1987 and 2007[^12^](https://www.zotero.org/google-docs/?whuXJo).

Unless otherwise stated, the autosomal genotypes of kidney failure patients and controls were imputed using either the Haplotype Reference Consortium r1.1 2016 on the Sanger imputation server, with phasing using Eaglev2.4. All data was then filtered for standard QC parameters (supplementary materials section on Data QC and processing).

Seven of these cohorts consisted of kidney transplant recipients (GECKO-T, DeKAF, GEN03, FRCBS, KiT-GENIE, QUB, TL, UKIRTC). 3 consisted of CKD patients with kidney failure (CanPREDDICT, GECKO, SKS) and 2 consisted exclusively of PKD patients (Mayo clinic PKD cohort, and IKGP). For the transplant cohorts where age of onset was not directly available, we used age of transplant minus 4 years for the North American cohorts and age of transplant minus 2 years for the European cohorts as a proxy for age of onset.

Other (39%) and unknown (11%) forms of primary renal disease were grouped together due to the heterogeneous nature of the other forms of primary renal disease. This group included, among other causes: hereditary nephropathy, Alport’s syndrome, congenital renal hypoplasia, Interstitial nephritis, congenital renal dysplasia, renal vascular disease, nephrocalcinosis, and kidney tumour.

## Summary statistics by primary renal disease

| **Variable** | **Overall** | **Glomerulonephritis** | **Other/Unknown** | **PKD** | **Type 2 Diabetes** |
| --- | --- | --- | --- | --- | --- |
| **Number of patients** | 10,826 | 1,756 | 5,891 | 2,850 | 1,329 |
| **Age of kidney failure, mean (range)** | 49 (0 - 95) | 45 (2 - 90) | 48 (0 - 94) | 52 (8 - 90) | 53 (19 - 95) |
| **Female, n (%)** | 4,522 (38) | 541 (31) | 2,203 (37) | 1,340 (47) | 438 (33) |

**Supplementary Table S1**: Summary statistics of all data, broken down by disease type. Age of kidney failure is in years and is death censored. Glomerulonephritis includes Glomerulonephritis and IgA nephropathy.

# Power analysis

We performed a power analysis using the *power.t.test* function from the package *stats in* R to determine the smallest effect size that could be reliably detected in the age of kidney failure. We calculated the number of non-missing patients along with the standard deviation of the age of onset. These were combined with a specification of 95% power and a significance level of 0.05 to generate a value for delta (the smallest effect size reliably detectable) using a two sample t-test power calculation.

The smallest difference in age of onset that this study is powered to detect at 95% power and 0.05 significance is 0.71 years (~8.5 months). The smallest difference in age of onset that the analysis looking at polygenic burden as a modifier of the primary PKD variant is powered to detect is 1.3 years (~15 months).

# Data QC and processing

The following criteria were used to determine inclusion in this study:

1. Standard QC parameters of minor allele frequency of 0.02, missingness 0.05, genotyping rate 0.05.
2. All participants were unrelated up to and including the level of 3^rd^ degree. This was determined using KING[^13^](https://www.zotero.org/google-docs/?eUbQsn).
3. All participants must be of European ancestry. This was determined using principal components analysis (PCA) with the reference European ancestry population from 1000 Genomes.

We used the PRSice2 software with a p-value threshold of 0.5 and physical distance threshold for clumping of 250 kb and LD threshold of 0.1 to generate the PRSs.

#

# Details of GWAS used to generate PRS

| **Trait** | **Population** | **Study** | **Discovery sample size** | **Ancestry** | **Validation cohort** | **SNP based heritability (%)** | **Effect size** | **Loci** |
| --- | --- | --- | --- | --- | --- | --- | --- | --- |
| Clinical Microalbuminuria (UACR > 30 mg/g) | CKDGen | Teumer et al. 2019[^14^](https://www.zotero.org/google-docs/?E4qmRz) | 564,257 | European | UKB | 4.3 | OR of 1.69 for quartile 4 vs 1, p=3×10^−191^ | 68 |
| eGFR | CKDGen | Wuttke et al. 2019[^15^](https://www.zotero.org/google-docs/?0zaufQ) | 567,460 | European | MVP | 7.1 | OR of chronic renal failure per 10% lower GRS 2.13, p=8.1×10^-38^ | 264 |
| Rapid eGFR decline of > 5 ml/min per 1.73 m^2^ per year | CKDGen + UKB | Gorski et al. 2021[^16^](https://www.zotero.org/google-docs/?iUf2Rj) | 19,901 cases, 175,244 controls | All | NA | NA | NA | 7 |
| Kidney Volume (KV) | UKB | Liu et al. 2021[^17^](https://www.zotero.org/google-docs/?IX5ECz) | 32,860 | White British | NA | 3.1 | NA | 9 |
| Hypertension | UKB | Wenjian et al. 2020[^18^](https://www.zotero.org/google-docs/?w5rttr) | 76,566 cases, 206,305 controls | White British | NA | NA | NA | 204 |

**Supplementary Table S2**: Details of GWAS used to generate PRS. Sample sizes and reference study for each trait for which a PRS was generated. The number of genome-wide significant loci found in each study is also given. UKB: UK Biobank, CKDgen: CKD genetic consortium, MVP: Million Veterans Programme. In our analysis, signs were flipped in the KV and eGFR PRSs in order that a higher PRS would be associated with a negative outcome (i.e. reduced eGFR and reduced KV).

# Validation of PRS methods

**Methods**

PRS for the same 5 traits relating to kidney function were also calculated using lassosum[^19^](https://www.zotero.org/google-docs/?WRzFZT). We used all the default parameters for lassosum including an n of 6000. We then got correlations between each of the scores computed using lassosum and PRSice.

**Results**

Note that these correlation coefficients are of a very similar magnitude to those found in a 2020 study that compared correlation of PRS for kidney function as calculated using PRSice and lassosum and found a correlation coefficient of 0.85[^20^](https://www.zotero.org/google-docs/?dFQk1Q).

| **PRS** | **Correlation coefficient** |
| --- | --- |
| Albuminuria | 0.75 |
| eGFR | 0.88 |
| Rapid eGFR decline | 0.81 |
| Hypertension | 0.84 |
| KV | 0.68 |
| **Average** | **0.79** |

**Supplementary Table S3**: Correlation between PRSs as calculated using lassosum and PRSice.

# PRS as a modifier of PKD primary mutation

| **Variable** | **Overall** | ***PKD1* Truncating** | ***PKD1* Non-truncating** |
| --- | --- | --- | --- |
| **Number of patients, n (%)** | 1,370 | 1,013 (74) | 357 (26) |
| **Age of kidney failure (years), mean (range)** | 51 (19 - 84) | 50 (19 - 81) | 53 (29 - 79) |
| **Female, n (%)** | 683 (50) | 511 (50) | 172 (48) |
| **Group, n (%)** |  |  |  |
| Mayo | 1,240 (91) | 919 (91) | 321 (90) |
| IKGP | 130 (10) | 94 (9) | 36 (10) |

**Supplementary Table S4**: Summary statistics of PKD patients where the diagnostic variant was known split by variant type.

| ***Characteristic*** | ***HR*** | ***p-value*** | ***95% CI*** |
| --- | --- | --- | --- |
| Diagnostic variant type |  |  |  |
| *PKD1-T* | *1* | *-* |  |
| *PKD1-NT* | ***0.76*** | ***<0.001*** | *0.67 - 0.86* |
| Sex |  |  |  |
| *Female* | *1* | *-* |  |
| *Male* | ***1.33*** | ***<0.001*** | 1.19 - 1.47 |
| Hypertension PRS |  |  |  |
| *Low burden* | 1.04 | 0.69 | *0.85 - 1.23* |
| *Intermediate burden* | 1 | - |  |
| *High burden* | 0.98 | 0.84 | *0.81 - 1.18* |
| Albuminuria PRS |  |  |  |
| *Low burden* | 1.09 | 0.36 | *0.90 - 1.32* |
| *Intermediate burden* | 1 | - |  |
| *High burden* | 0.98 | 0.83 | *0.83 - 1.15* |
| Low eGFR PRS |  |  |  |
| *Low burden* | 0.97 | 0.71 | *0.81 - 1.15* |
| *Intermediate burden* | 1 | - |  |
| *High burden* | 0.94 | 0.56 | *0.77 - 1.15* |
| Low KV PRS |  |  |  |
| *Low burden* | 1.19 | 0.08 | *0.98 - 1.45* |
| *Intermediate burden* | 1 | - |  |
| *High burden* | 1.05 | 0.58 | *0.88 - 1.25* |
| Rapid eGFR decline PRS |  |  |  |
| *Low burden* | 1.07 | 0.2 | *0.65 - 1.77* |
| *Intermediate burden* | 1 | - |  |
| *High burden* | 0.92 | 0.81 | *0.48 - 1.78* |

**Supplementary Table S5**: Multivariate cox PH models for age of kidney failure controlling for PRS, sex, and primary mutation (in Mayo and IKGP cohorts).

# Bibliography

[1. Levin A, Rigatto C, Brendan B, et al. Cohort profile: Canadian study of prediction of death, dialysis and interim cardiovascular events (CanPREDDICT). *BMC Nephrology*. 2013;14(1):1-11. doi:10.1186/1471-2369-14-121](https://www.zotero.org/google-docs/?jCx2TY)

[2. Lanktree MB. GECKO and GECKO-Transplant Studies. Published 2023. http://www.nephrogenetics.ca/research.html](https://www.zotero.org/google-docs/?jCx2TY)

[3. Matas AJ, Fieberg A, Mannon RB, et al. Long-term follow-up of the DeKAF cross-sectional cohort study. *American Journal of Transplantation*. 2019;19(5):1432-1443. doi:10.1111/ajt.15204](https://www.zotero.org/google-docs/?jCx2TY)

[4. Mohamed ME, Schladt DP, Guan W, et al. Tacrolimus Troughs and Genetic Determinants of Metabolism in Kidney Transplant Recipients: A comparison of four ancestry groups. *American Journal of Transplantation*. 2019;19(10):2795-2804. doi:10.1111/ajt.15385](https://www.zotero.org/google-docs/?jCx2TY)

[5. Markkinen S, Helanterä I, Lauronen J, Lempinen M, Partanen J, Hyvärinen K. Mismatches in Gene Deletions and Kidney-related Proteins as Candidates for Histocompatibility Factors in Kidney Transplantation. *Kidney International Reports*. 2022;7(11):2484-2494. doi:10.1016/j.ekir.2022.08.032](https://www.zotero.org/google-docs/?jCx2TY)

[6. Garrigue V, Szwarc I, Giral M, et al. Influence of anemia on patient and graft survival after renal transplantation: Results from the French DIVAT Cohort. *Transplantation*. 2014;97(2):168-175. doi:10.1097/TP.0b013e3182a94a4d](https://www.zotero.org/google-docs/?jCx2TY)

[7. Lavu S, Vaughan LE, Senum SR, et al. The value of genotypic and imaging information to predict functional and structural outcomes in ADPKD. *JCI Insight*. 2020;5(15):1-18. doi:10.1172/jci.insight.138724](https://www.zotero.org/google-docs/?jCx2TY)

[8. Steers NJ, Li Y, Drace Z, et al. Genomic Mismatch at LIMS1 Locus and Kidney Allograft Rejection . *New England Journal of Medicine*. 2019;380(20):1918-1928. doi:10.1056/nejmoa1803731](https://www.zotero.org/google-docs/?jCx2TY)

[9. Benson KA, Murray SL, Senum SR, et al. The genetic landscape of polycystic kidney disease in Ireland. *European Journal of Human Genetics*. 2021;29(5):827-838. doi:10.1038/s41431-020-00806-5](https://www.zotero.org/google-docs/?jCx2TY)

[10. Tollitt J, Odudu A, Flanagan E, Chinnadurai R, Smith C, Kalra PA. Impact of prior stroke on major clinical outcome in chronic kidney disease: the Salford kidney cohort study. *BMC Nephrol*. 2019;20(1):432. doi:10.1186/s12882-019-1614-5](https://www.zotero.org/google-docs/?jCx2TY)

[11. Eisenga MF, Gomes-Neto AW, Van Londen M, et al. Rationale and design of TransplantLines: A prospective cohort study and biobank of solid organ transplant recipients. *BMJ Open*. 2018;8(12):1-13. doi:10.1136/bmjopen-2018-024502](https://www.zotero.org/google-docs/?jCx2TY)

[12. Hernandez-Fuentes MP, Franklin C, Rebollo-Mesa I, et al. Long- and short-term outcomes in renal allografts with deceased donors: A large recipient and donor genome-wide association study. *American Journal of Transplantation*. 2018;18(6):1370-1379. doi:10.1111/ajt.14594](https://www.zotero.org/google-docs/?jCx2TY)

[13. Manichaikul A, Mychaleckyj JC, Rich SS, Daly K, Sale M, Chen WM. Robust relationship inference in genome-wide association studies. *Bioinformatics*. 2010;26(22):2867-2873. doi:10.1093/bioinformatics/btq559](https://www.zotero.org/google-docs/?jCx2TY)

[14. Teumer A, Li Y, Ghasemi S, et al. Genome-wide association meta-analyses and fine-mapping elucidate pathways influencing albuminuria. *Nature Communications*. 2019;10(1). doi:10.1038/s41467-019-11576-0](https://www.zotero.org/google-docs/?jCx2TY)

[15. Wuttke M, Li Y, Li M, et al. A catalog of genetic loci associated with kidney function from analyses of a million individuals. *Nature Genetics*. 2019;51(6):957-972. doi:10.1038/s41588-019-0407-x](https://www.zotero.org/google-docs/?jCx2TY)

[16. Gorski M, Jung B, Li Y, et al. Meta-analysis uncovers genome-wide significant variants for rapid kidney function decline. *Kidney International*. 2021;99(4):926-939. doi:10.1016/j.kint.2020.09.030](https://www.zotero.org/google-docs/?jCx2TY)

[17. Liu Y, Basty N, Whitcher B, et al. Genetic architecture of 11 organ traits derived from abdominal MRI using deep learning. *eLife*. 2021;10:1-30. doi:10.7554/eLife.65554](https://www.zotero.org/google-docs/?jCx2TY)

[18. Bi W, Fritsche LG, Mukherjee B, Kim S, Lee S. A Fast and Accurate Method for Genome-Wide Time-to-Event Data Analysis and Its Application to UK Biobank. *Journal of Cleaner Production*. 2020;107(2):222-233. doi:10.1016/j.ajhg.2020.06.003](https://www.zotero.org/google-docs/?jCx2TY)

[19. Mak TSH, Porsch RM, Choi SW, Zhou X, Sham PC. Polygenic scores via penalized regression on summary statistics. *Genetic Epidemiology*. 2017;41(6):469-480. doi:10.1002/gepi.22050](https://www.zotero.org/google-docs/?jCx2TY)

[20. Yu Z, Jin J, Tin A, et al. Polygenic Risk Scores for Kidney Function and Their Associations with Circulating Proteome, and Incident Kidney Diseases. *JASN*. 2021;32(12):3161-3173. doi:10.1681/ASN.2020111599](https://www.zotero.org/google-docs/?jCx2TY)
